# The Future of Unqualified Nurses

**Published:** 1919-08-30

**Authors:** 


### THE MATRONS' AND SISTERS' DEPARTMENT.

### THE FUTURE OF UNQUALIFIED NURSES.

By general consent the problem of the partly trained nurse has been allowed to fall into the background during the discussions on registration. The importance of having one portal for trained nurses is universally recognised. All agree that there must be a minimum standard of education and professional knowledge which all who are stamped with the hall-mark of the professional nurse must attain. Is there, however, any one so simple as to suppose that the establishment of a registration board will suffice under a voluntary system to extinguish the partly trained nurse? It will be much, and very much, if trained nurses can be generally shepherded within the fold of registration, so that the public may clearly differentiate between women who know their business and women who only pretend to be "just as good." But do we not all know that the "just as good" people will be there all the same after registration, and that they will continue to be manufactured and multiplied for reasons which are outside the control of any system of registration? It becomes a matter of some urgency to consider how best they may be dealt with in the interests of the public.

Suppose that all partly trained women, all attendants, for instance, and women trained exclusively in one branch of the profession, all persons "trained" in tiny institutions, all the probationers who have failed to complete their course and gain a certificate, all the cottage nurses, all, in short, who have some knowledge of nursing but are not eligible for registration were prohibited from practice, is it not quite clear that very great hardship to the public would ensue? Some of those whom we have enumerated would perhaps be admitted to registration during the time of grace, but when this is over the causes which contribute to bring the partly trained nurse into existence will still be operating. We are convinced that whatever steps may be taken to eliminate her from our midst, she will be always with us, because there is a place for her in the great network of agencies concerned with the sick.

It is inconceivable that the Health Ministry will not sooner or later find itself under the necessity of getting control over the immense company of women who earn their living by nursing, yet have no recognised qualifications. Real organisation of nursing affairs in this country means recognition of the fact that people who undertake "habitually and for gain" the nursing of the sick, fall into two definite categories: nursing sisters hereafter to be registered by the State, and—the others. To make elaborate rules for the regulation of highly qualified women and leave the others to follow their own sweet will must in the end prove highly unsatisfactory. While the authorities are stoutly declaring that there must be only one door to the nursing profession, we are edified by the spectacle of a back-door wide open and with crowds pouring in, not merely unopposed, but aided by private and by State funds. The work of training nurses accomplished by the County Nursing Association is freely aided by grants from public funds. Does any one suppose that these partly State-trained nurses receive a three-hospital-year course? Notoriously, they are trained merely for some six months in midwifery, and for another six months in district work among the poor. There are thousands of them transforming the face of the country by their patient labours. Who will suggest that they be denied the name of nurse? But there is nothing whatever to prevent them, after fulfilling their contract, from entering the ranks of private nursing and obtaining work under doctors with whom they have become acquainted in the course of their district work. Are registered nursing sisters to be liable to penalties for unprofessional conduct while half or quarter-trained women are suffered, even perhaps after glaring blunders, to represent themselves as competent to undertake any kind of case? This is such manifest injustice as must, unless due care be taken, render registration abortive.

No one wants to penalise the women whose training comes short of the minimum. But in the public interest they ought to be licensed for such work and for such work only, as they are capable of performing. They ought to be inspected and controlled. Such a course would not open a back-door to the profession. It would merely open the eyes of the authorities to the fact that the back-door already exists and cannot be closed.

